# Effects of Pemafibrate and Eicosapentaenoic Acid Ethyl Ester on Endothelial Function in Patients With Hypertriglyceridemia and Coronary Artery Disease: A Study Protocol for a Multicenter, Open-Label Randomised Controlled Trial

**DOI:** 10.7759/cureus.81104

**Published:** 2025-03-24

**Authors:** Trou Miyoshi, Yasushi Matsuzawa, Masayuki Doi, Shinsuke Yuasa, Seigo Sugiyama

**Affiliations:** 1 Cardiovascular Medicine, Okayama University, Okayama, JPN; 2 Cardiovascular Medicine, Kumamoto University, Kumamoto, JPN; 3 Cardiology, Kagawa Prefectural Central Hospital, Takamatsu, JPN; 4 Cardiovascular Medicine, Jinnouchi Hospital, Kumamoto, JPN

**Keywords:** cardiovascular risk, eicosapentaenoic acid, endothelial function, pemafibrate, triglyceride

## Abstract

Despite intensive low-density lipoprotein cholesterol-lowering therapies effectively reducing cardiovascular events, residual cardiovascular risks remain significant, with hypertriglyceridemia being an important contributing factor. Pemafibrate, a novel selective peroxisome proliferator-activated receptor alpha modulator, has shown strong triglyceride-lowering effects and potential vascular benefits. Similarly, eicosapentaenoic acid ethyl ester (EPA) has demonstrated cardiovascular protective effects, particularly in patients with hypertriglyceridemia. However, the comparative impact of these agents on endothelial function, a key marker of atherosclerotic progression, has not been thoroughly evaluated in patients with coronary artery disease (CAD). The PRIME (PRospective comparIson of peMafibrate and Eicosapentaenoic acid ethyl ester on vascular functions for hypertriglyceridemia) trial is a multi-center, open-label, randomised trial designed to compare the effects of pemafibrate and EPA on endothelial function in patients with CAD and hypertriglyceridemia. Patients receiving statin therapy with fasting triglyceride levels ≥150 mg/dL will be randomised into two groups: pemafibrate (0.2 mg/day, with possible dose escalation to 0.4 mg/day) or EPA (1800 mg/day, with possible dose escalation to 2700 mg/day). Endothelial function will be assessed with reactive hyperemia index (RHI). The primary endpoint is the change in RHI at 12 weeks. The secondary endpoints include the changes in RHI at 24 weeks, correlations between changes in RHI and changes in lipid biomarkers, and changes in biochemical parameters at 12 and 24 weeks. This study investigates the comparative effects of pemafibrate and EPA on endothelial function, addressing an unmet need in managing residual cardiovascular risk in patients with CAD. The findings will contribute to the optimisation of treatment strategies in patients with CAD and hypertriglyceridemia.

## Introduction

Residual cardiovascular risks remain a major concern in patients with coronary artery disease (CAD), even when low-density lipoprotein cholesterol (LDL-C) is optimally management with statins [1]. Among these residual risks, hypertriglyceridemia is recognised as a major contributor to cardiovascular events [2]. The subanalysis of the PROVE IT-TIMI (Pravastatin or Atorvastatin Evaluation and Infection Therapy-Thrombolysis In Myocardial Infarction) 22 trial demonstrated that patients after acute coronary syndrome who were on statin treatment with triglyceride (TG) level < 150mg/dL showed significantly lower coronary heart disease risk with a hazard ratio of 0.80 (95% confidence interval (CI): 0.66-0.97) [3]. Several genetic studies also demonstrated taut elevated TG-rich lipoproteins were causally associated with atherosclerotic cardiovascular disease [4,5].

Pharmacological interventions targeting TG levels are garnering attention [6]. Fibrate and eicosapentaenoic acid ethyl ester (EPA), an omega-3 fatty acid, have gained prominence in this context. Pemafibrate is novel selective peroxisome proliferator-activated receptor-α modulator [7,8]. It has potent TG-lowering effects, as well as anti-inflammatory and lipid-modulating properties. For instance, an animal study demonstrated that pemafibrate suppressed coronary stent-induced cellular inflammation and neointima expression of tumor necrosis factor-α [9]. A clinical study demonstrated that the administration of pemafibrate for four weeks enhanced reverse cholesterol transport in high-density lipoprotein [10]. EPA also has TG-lowering and anti-inflammatory properties. Our previous study showed that oral administration of EPA increased plasma levels of 18-hydroxyeicosapentaenoic acid, an anti-inflammatory mediator [11].

However, the findings of drug intervention trials for TG aimed at preventing the development of cardiovascular disease have been inconclusive. In fibrate trials, a study with post-myocardial infarction patients given both fibrate and niacin showed that ischaemic heart disease mortality was reduced by 36%. On the other hand, a recent study including over 10,000 patients with diabetes and mild-to-moderate hypertriglyceridemia who received pemafibrate did not show a significant reduction in cardiovascular events [12]. Meanwhile, high-dose omega-3 fatty acid supplementation showed no decrease in the incidence of cardiovascular events despite a 20% decrease in TG levels [13], whereas, in a trial of EPA, a significant reduction of cardiovascular events was observed in the setting of secondary prevention of cardiovascular disease risk [14].

Endothelial dysfunction is an early indicator of atherosclerosis and an independent predictor of cardiovascular outcomes. Our previous study demonstrated that a fibrate improved flow-mediated dilation (FMD) in patients with metabolic syndrome [15] and that oral EPA improved FMD in patients with CAD [16]. Despite the established TG-lowering capabilities of both agents, a direct comparative evaluation of their effects on endothelial function has not been conducted. Reactive hyperemia peripheral arterial tonometry (RH-PAT) offers a non-invasive and reliable measure of endothelial function [17]. The RH-PAT data is automatically analysed by a computer in an operator-independent manner which is a major advantage compared to FMD in the brachial artery. In addition, a robust test-retest repeatability of reactive hyperemia index (RHI) has been reported, suggesting that RHI may be useful in assessing the effect of treatment on endothelial function [18]. Studies have shown that improvements in endothelial function correlate with reductions in cardiovascular risk [19].

The PRIME (PRospective comparIson of peMafibrate and Eicosapentaenoic acid ethyl ester on vascular functions for hypertriglyceridemia) trial described here is designed to compare the effects of pemafibrate and EPA on endothelial function in patients with CAD and hypertriglyceridemia. The objective of this trial is to establish the superiority of pemafibrate in improving endothelial function in patients with CAD and hypertriglyceridemia, compared to EPA. The findings of this study will provide clinically relevant insights that may guide therapeutic decision-making in managing residual cardiovascular risks.

## Materials and methods

Study design

The PRIME trial is a multi-center, prospective, open-labeled, parallel-group, randomised controlled trial to assess the comparative effects of pemafibrate and EPA on endothelial function using RH-PAT index (RHI) in patients with CAD and hypertriglyceridemia (trial registration number: jRCTs061200010).

Participants

The study is designed to include a total of 100 participants, with 50 individuals assigned to each group. The patients were recruited between September 2021 and September 2024.

Inclusion Criteria

The inclusion criteria for this study require patients to have been on statin therapy for at least 12 weeks before consent and to have fasting TG levels of ≥150 mg/dL within eight weeks prior to consent. Eligible patients must have a history of coronary revascularisation procedures, such as percutaneous coronary intervention (PCI) or coronary artery bypass grafting (CABG), and a clinically diagnosed ischemic heart disease confirmed by imaging techniques like coronary CT, coronary angiography, or myocardial scintigraphy. Participants must be aged 20 years or older and capable of understanding and complying with study procedures, providing written informed consent.

Exclusion Criteria

Exclusion criteria include renal insufficiency, defined as serum creatinine over 2.5 mg/dL, and severe liver dysfunction, with aspartate aminotransferase (AST) or alanine transaminase (ALT) levels over 100 IU/L. Patients with contraindications to pemafibrate and EPA are also excluded. Contraindications to pemafibrate include a history of allergy to pemafibrate, severe hepatic impairment, cirrhosis with Child-Pugh B or C, biliary obstruction, cholelithiasis, pregnancy or potential pregnancy, and the use of cyclosporine or rifampicin. Contraindications for EPA include active bleeding. Additionally, patients with a history of acute coronary syndrome within three months prior to consent, a history of PCI or CABG within three months prior to informed consent, or a planned PCI or CABG after informed consent are excluded.

Other exclusion criteria include the use of fibrates or omega-3 fatty acids within 12 weeks prior to informed consent, the use of phosphodiesterase-5 inhibitors, fasting TGs over 500 mg/dL within 12 weeks prior to informed consent, and failure to meet LDL-cholesterol management criteria (>100 mg/dL, based on Japan Atherosclerosis Society Guidelines for Prevention of Atherosclerotic Cardiovascular Diseases 2017) within 12 weeks prior to informed consent. Patients with secondary dyslipidemia, including drug-induced dyslipidemia, or primary dyslipidemia, including familial hypercholesterolemia, are also excluded. Furthermore, exclusion criteria include alcoholism, diabetes mellitus with poor control (hemoglobin A1c >9.5%) or type 1 diabetes mellitus, vasospastic angina and microvascular angina, and a life expectancy of fewer than six months.

Study procedures and randomisation

**Figure 1:**
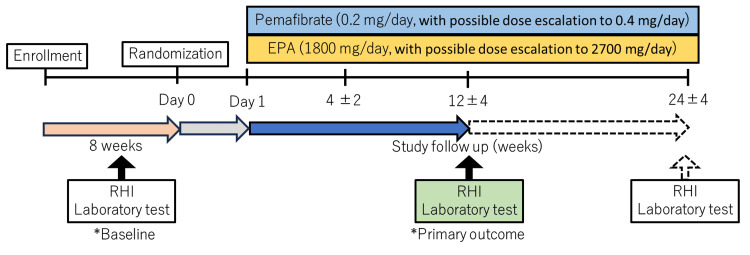
Study design. The arrows depict the patient flow and the schedule for follow-up visits. Patients diagnosed with triglyceridemia and coronary artery disease are assessed to confirm that their triglyceride levels are 150 mg/dL or higher. Those who meet the inclusion criteria will receive one of the study drugs within one day of randomisation (indicated by the grey arrow). Following drug administration, there is a required follow-up period lasting 12 weeks (shown with a blue arrow). After the 12-week period, patients who provide consent for continued monitoring will enter an extended follow-up phase (indicated by the dotted arrow). During this phase, any modifications to the assigned medication are prohibited. EPA: eicosapentaenoic acid; RHI: reactive hyperemia index

The flowchart of the study is illustrated in Figure 1. Patients fulfilling all criteria and who will provide written informed consent to participate in this study will be randomly assigned (1:1) to the pemafibrate group (0.1 mg twice daily, with dose adjustments up to 0.2 mg twice daily) or EPA group (900 mg administered two to three times daily). The aim of dose escalation is to achieve a fasting TG level of <150 mg/dL; however, this depends on the patient's response and tolerance. Randomisation will be performed using a computer-generated random sequence web response system by central randomisation. The patients will be stratified by age (<65 years, ≥65 years), sex (male, female), baseline TG levels (<250 mg/dL, ≥250 mg/dL), and baseline RHI (<1.5, ≥1.5).

Assessments to be performed during the study period are presented in Table 1. At the baseline, 12 weeks, and 24 weeks, the same data will be collected, while baseline assessments will include demographic data and medication history. Endothelial function will be assessed using RH-PAT (Endo-PAT2000; Itamar Medical, Caesarea, Israel). The follow-up at four weeks will be performed at four weeks for safety monitoring. Safety assessments will include adverse event monitoring and regular laboratory tests for hepatic and renal functions. Medication adherence is monitored by verbal confirmation at the time of the visit. As the RHI is affected by exercise and medication, no major lifestyle changes are allowed during the study period, and changes in medication are prohibited.

**Table 1:** Assessments during the study period.

Assessment	Enrollment	Study follow-up period			Discontinuation (at any time)
		4 weeks	12 weeks (±4 weeks)	24 weeks (±4 weeks)	
Medical examination	×	×	×	×	×
Written informed consent	×				×
Clinical symptom	×	×	×	×	×
Adverse events		×	×	×	×
Treatment discontinuation		×	×	×	×
Vital signs	×	×	×	×	×
Blood pressure	×	×	×	×	×
Body weight	×		×	×	×
Laboratory tests	×	×	×	×	×
Reactive hyperemia index	×		×	×	×

RH-PAT

The principle of RH-PAT has been described previously [20]. This technique provides a noninvasive assessment of blood volume fluctuations associated with pulse waves in the fingertip. Caffeine intake and smoking are prohibited before the test. Without knowing the treatment assignment, the measurement is performed in the fasting state. To perform the measurement, a blood pressure cuff is applied to one upper arm, while the opposite arm serves as a control. Sensors for PAT monitoring are placed on the finger of each hand. After a five-minute stabilisation period, the cuff is inflated to a pressure of 60 mmHg above the systolic level or up to 200 mmHg for five minutes before being released to elicit reactive hyperemia. The RHI quantifies the degree of hyperemic response and is automatically computed by specialised software. This calculation is based on the ratio of the mean PAT signal amplitude over a one-minute window, starting 1.5 minutes post-deflation (occluded arm, C; control arm, A), to the mean amplitude recorded during the 2.5-minute baseline period preceding cuff inflation (occluded arm, D; control arm, B). The RHI is determined using the formula (C/D)/(A/B). The analysis is fully automated, ensuring consistency and minimising operator bias. Previous studies have demonstrated that RH-PAT technology has excellent reproducibility [18,21-23].

Measurements of biochemistries

Fasting blood samples will be drawn from the antecubital vein in the early morning using standard phlebotomy procedures without knowing the treatment allocation. The blood samples taken at baseline, 12, and 24 weeks include TG, LDL-cholesterol, high-density lipoprotein cholesterol levels, small dense LDL-C level, high-sensitive C-reactive protein, liver function indices, apolipoproteins (apolipoprotein A-I, apolipoprotein B, apolipoprotein C-III, apolipoprotein, remnant like protein-C), malondialdehyde-modified low-density lipoprotein, interleukin-1β, tumor necrosis factor-α, fibrinogen, and hemoglobin A1c, estimated glomerular filtration rate, and fatty acid fractionation. Urinary 8-hydroxy-2'-deoxyguanosine is also collected. The blood samples taken at four weeks include TG, LDL-cholesterol, high-density lipoprotein cholesterol levels, liver function indices, and estimated glomerular filtration rate. All biomarker levels will be analysed at the core laboratory (SRL Corp., Ltd., Tokyo, Japan).

Outcomes

Table 2 presents the primary and secondary outcomes of this study.

**Table 2:** Primary and secondary outcomes. RHI: reactive hyperemia index; EPA: eicosapentaenoic acid; LDL: low-density lipoprotein; ln: logarithmic

Endpoints	Details
Primary	RHI change after 12 weeks of treatment between the pemafibrate and EPA groups, defined as the difference in logarithmic RHI change calculated as follows: The difference of lnRHI change = ([lnRHI at follow-up - lnRHI at baseline] in the pemafibrate group)-([lnRHI at follow-up - lnRHI at baseline] in the EPA group)
Secondary	1. The difference in RHI change after 24 weeks of treatment between the pemafibrate and EPA groups
	2. Association between RHI and biochemical parameters including triglyceride, LDL-cholesterol, and high-density lipoprotein cholesterol levels, small dense LDL-cholesterol level, high-sensitive C-reactive protein level at baseline and 12 weeks
	3. The differences in change in the following parameters between the pemafibrate and EPA groups: triglyceride, LDL-cholesterol, and high-density lipoprotein cholesterol levels, small dense LDL-cholesterol level, high-sensitivity C-reactive protein level

The primary outcome of the study was to compare the improvement in endothelial function between the pemafibrate and the EPA groups. The secondary outcomes were to assess the change in lipid profiles between the pemafibrate and the EPA groups, to determine correlations between endothelial function and biochemical parameters, and to assess safety.

Further exploratory analysis will include levels of apolipoproteins (apolipoprotein A-I, apolipoprotein B, apolipoprotein C-III, apolipoprotein, remnant-like protein-C), malondialdehyde-modified low-density lipoprotein, interleukin-1β, tumor necrosis factor-α, fibrinogen, urinary 8-hydroxy-2'-deoxyguanosine, and hemoglobin A1c, estimated glomerular filtration rate, and fatty acid fractionation. The exploratory outcomes are hypothesis-generating.

Safety outcomes

Safety will be evaluated in accordance with the Common Terminology Criteria for Adverse Events (CTCAE) version 5.0 guidelines. Any adverse events (e.g., bleeding, acute pancreatitis, severe liver dysfunction, deterioration of renal function), whether spontaneously reported by patients or identified by investigators, will be thoroughly documented. The records will include details such as the timing, clinical manifestations, management strategies, duration, resolution, and potential association with the administered medication. Causality assessment is performed by investigator judgment. For cases involving abnormal laboratory findings, patient monitoring will continue until the results normalise or are determined to be unrelated to the assigned treatment. If a participant becomes pregnant, fetal outcomes will be monitored.

Study oversight and organisation

The Steering Committee members, who were also responsible for designing the study, will supervise its implementation. Any significant adverse events occurring within 30 days following the final administration of the study drug, or beyond this period if there is a suspected link to the drug, as well as any reported pregnancies, will be promptly communicated by the investigator to both the Steering Committee and the sponsor in compliance with good clinical practice.

Discontinuation of the study for individual participants

The principal investigator or co-investigator may discontinue the study for an individual participant if they determine that it is impossible to continue the study for the participant due to any of the following reasons. The criteria for discontinuation and withdrawal are as follows: adverse events that are suspected to be caused by the study drug and that make continuation impossible (a severity of grade 4 or higher according to the CTCAE guideline), a new cardiovascular event, the participant refuses to continue or withdraws consent, and if pregnancy is confirmed.

Statistical analysis

Sample Size Calculation

No study has investigated the changes in RHI in response to fibrates for hypertriglyceridemia treatment. However, two studies have reported a link between increased TG levels and decreased RHI, with a decrease of approximately 50 mg/dL resulting in a reduction of approximately 0.08 in lnRHI [24,25]. In addition, previous reports have examined changes in FMD, another indicator of endothelial function [15-17,19,26]. Based on these studies, a 10% reduction in TG levels has been estimated to result in a 0.4% increase in FMD. The standard deviation of FMD measurements in multicenter studies is approximately 1.3%. Assuming reductions in TG levels of 45% and 20% for the pemafibrate and EPA groups, respectively, the expected increases in FMD are 1.8% and 0.8%, with a difference of 1.0% between the two groups. When this difference was converted to a logarithmic RHI (lnRHI, as RHI follows a non-normal distribution), the difference in lnRHI between the pemafibrate and EPA groups was estimated to be 0.076, with a standard deviation of 0.11. Assuming a 25% difference in TG reduction between the EPA and the pemafibrate groups in the present study, this would equate to a 50 mg/dL reduction at a baseline of 200 mg/dL, which is in close agreement with the value predicted by the FMD and is reasonable. Using a two-sample t-test with a two-sided significance level of 5% and a statistical power of 90%, the required sample size was calculated to be 90 participants (45 in each group). Considering the possibility of dropouts during the study period, the target number of patients was set at 100 in both groups (50 in each group). Assuming a statistical power of 80%, the number of cases required would be 66, which means that even if more than 10% of cases were to be excluded, the analysis would still be possible.

Analysis Plan

The efficacy analysis will be conducted on the full analysis set (FAS), which comprises all randomised patients who have received one dose of the study drug and undergone at least one follow-up assessment. Patients lacking RHI data or those who discontinue or withdraw from treatment will not be included in the FAS. For any missing values at the 24-week mark, the last observed value for the respective variable will be carried forward (last observation carried forward). Furthermore, a baseline observation carried forward approach will be utilised in the primary outcome analysis. The analysis will be performed based on the intention-to-treat principle, considering patients in their originally assigned treatment groups. Sensitivity analysis will be carried out on data corrected for multiple imputations.

The primary outcome will be evaluated in the FAS using an analysis of covariance (ANCOVA), with a significance level set at α = 0.05, to analyse the ratio of the RHI change rate. RHI has a skewed distribution. Therefore, the logarithmic transformation of RHI is used for analysis. The ratio of the RHI change rate at 12 weeks between the two groups will be assessed. Adjusted covariates will include the assigned treatment (pemafibrate, EPA), age (<median, ≥median), sex (male, female), and baseline TG levels (<median value, ≥median value) as stratified factors of randomisation. Furthermore, the RHI change rate, the ratio of the RHI change rate and 95% CIs will be calculated.

The secondary outcomes, the difference in RHI change at 24 weeks and each lipid parameter at 12 and 24 weeks between the pemafibrate and EPA groups separately, will be analysed using ANCOVA as the primary outcome. The association between changes in RHI and changes in lipid parameter levels at baseline, 12, and 24 weeks will be analysed using Pearson’s analysis.

Prespecified subgroup analyses will be performed on the primary outcome according to the following subgroups: age (<median, ≥median), sex (male, female), baseline TG levels (<median, ≥median), baseline RHI (<median, ≥median), estimated glomerular filtration rate (>60 mL/min/1.73 m^2^, <60 mL/min/1.73 m^2^), body weight (<median, ≥median), hemoglobinA1c (<median, ≥median), presence of current or past smoking. P-values for interaction will not be calculated.

The safety analysis will focus primarily on the safety analysis population (SAFETY), which includes all patients who receive at least one dose of the study drug. Patients who withdraw before receiving any dose will not be included in SAFETY, while those who withdraw for other reasons will remain part of the analysis. This safety evaluation will use an as-treated methodology, analysing data based on the actual treatment received. The assessment of adverse events will be performed using logistic analysis.

Following amendments to the regulations under the Infectious Diseases Act in May 2023, medical restrictions in Japan have been relaxed. This change will be analysed through a sensitivity study to determine whether there are any differences in primary outcomes in the population before and after this amendment.

The planned comparisons are predefined and analyses are performed using a two-way approach. A P-value of less than 0.05 will be used as the criterion for statistical significance. All statistical analyses will be performed using IBM SPSS Statistics for Windows, Version 25 (Released 2017; IBM Corp., Armonk, New York, United States). The statistical analysis plan will be developed jointly by the principal investigator and a biostatistician before patient recruitment is completed and the database is closed.

## Results

The trial began in September 2021; however, case recruitment was delayed due to the effects of the COVID-19 pandemic. As a result, the enrollment period was extended until September 2024. The study results will be available after the trial's completion. The results will be obtained once the study has been completed.

## Discussion

This study aims to provide critical insights into the comparative effects of pemafibrate and EPA on endothelial function in patients with CAD and hypertriglyceridemia. By addressing the lack of direct comparative data, the PRIME trial will contribute to the understanding of the role of these therapies in mitigating residual cardiovascular risks.

A notable strength of this study lies in its focus on endothelial function, assessed through the RHI, as the primary endpoint. Endothelial dysfunction is a well-recognised precursor of atherosclerosis, and improvements in endothelial function have been associated with favorable cardiovascular outcomes [27]. The use of the RHI, a non-invasive and reproducible technique, in this study, ensures reliable measurements across multiple centers [19].

The study design also emphasises a pragmatic approach, enrolling a patient population reflective of real-world clinical settings. By including participants with confirmed CAD and hypertriglyceridemia who are already receiving statin therapy, the findings will have direct implications for managing residual cardiovascular risks in high-risk patients.

Despite its strengths, the study has potential limitations that must be acknowledged. First, the open-label design, although practical, may introduce bias. However, in this study, the use of RHI, a non-invasive and highly reproducible technique, ensures consistent and reliable measurements across multiple centers [19]. Second, the relatively short follow-up period may limit the assessment of long-term outcomes. However, we have reported that changes in RHI can be observed after treatment for three to 24 weeks [28,29]. In addition, a previous study showed that improvement of endothelial function after treatment for six months was associated with better prognosis in patients with CAD [27]. Taken together, we believe a 12-24 weeks observation period is appropriate. Third, RHI is not a direct predictor of hard cardiovascular outcomes, while a meta-analysis indicated that RHI significantly predicts cardiovascular events [30]. Further research is required to ascertain the extent to which improvements in RHI contribute to improved prognoses.

Nonetheless, this study will provide valuable insights into the comparative efficacy of pemafibrate and EPA on endothelial function in patients with CAD and hypertriglyceridemia. The findings may help guide the selection of one agent over the other for reducing residual cardiovascular risk.

## Conclusions

This study is expected to provide valuable insights into the comparative effects of pemafibrate and EPA on endothelial function in patients with CAD and hypertriglyceridemia. By addressing this critical aspect of residual cardiovascular risk, the results will help to optimise therapeutic strategies for high-risk patients. The study design, focusing on endothelial function as a marker of atherosclerosis, ensures relevance and clinical applicability. Despite some limitations, the results will improve our understanding of how these interventions affect vascular health. Ultimately, this research could pave the way for more effective risk reduction approaches in cardiovascular care.
